# Autoinhibition of the Ron receptor tyrosine kinase by the juxtamembrane domain

**DOI:** 10.1186/1478-811X-12-28

**Published:** 2014-04-16

**Authors:** Xin Wang, Neela Yennawar, Pamela A Hankey

**Affiliations:** 1 Graduate Program in Cell and Developmental Biology, The Pennsylvania State University, University Park, PA 16802, USA; 2 Huck Institutes for Life Sciences, The Pennsylvania State University, University Park, PA 16802, USA; 3 Department of Veterinary and Biomedical Sciences, The Pennsylvania State University, 115 Henning Building, University Park, PA 16802-3500, USA

**Keywords:** Ron, Receptor tyrosine kinase, Autoinhibition, Juxtamembrane domain

## Abstract

**Background:**

The Ron receptor tyrosine kinase (RTK) has been implicated in the progression of a number of carcinomas, thus understanding the regulatory mechanisms governing its activity is of potential therapeutic significance. A critical role for the juxtamembrane domain in regulating RTK activity is emerging, however the mechanism by which this regulation occurs varies considerably from receptor to receptor.

**Results:**

Unlike other RTKs described to date, tyrosines in the juxtamembrane domain of Ron are inconsequential for receptor activation. Rather, we have identified an acidic region in the juxtamembrane domain of Ron that plays a central role in promoting receptor autoinhibition. Furthermore, our studies demonstrate that phosphorylation of Y^1198^ in the kinase domain promotes Ron activation, likely by relieving the inhibitory constraints imposed by the juxtamembrane domain.

**Conclusions:**

Taken together, our experimental data and molecular modeling provide a better understanding of the mechanisms governing Ron activation, which will lay the groundwork for the development of novel therapeutic approaches for targeting Ron in human malignancies.

## Background

Dysregulated RTK activity is closely associated with a broad range of human malignancies. Thereby, understanding the complex mechanisms by which RTKs are regulated is of clinical significance due to potential applications for therapeutic intervention against cancers [1,2]. Overexpression or constitutive activation of the Ron receptor has been demonstrated to drive strong oncogenic phenotypes in cancers derived from breast, colon, lung, and prostate [3-5]. The oncogenic potential of Ron is represented by its capacity to induce migration, invasion, growth, survival and epithelial-mesenchamal transition of epithelial tumor cells, as well as to promote pro-tumoral activities of tumor-associated macrophages [6-13]. Inhibition of Ron expression using Ron specific siRNA dramatically decreases tumorigenic and invasive activities in colorectal carcinoma cells [14]. A human neutralizing antibody against Ron exhibits partial inhibition of colon, lung and pancreatic tumor growth in xenograft models [15], and a small molecule kinase inhibitor of Ron that has antitumor activity *in vivo* has been described [16]. Consequently, Ron is a promising target for therapeutic intervention against tumorigenic activities and malignant phenotypes [3,17,18].

**Figure 1:**
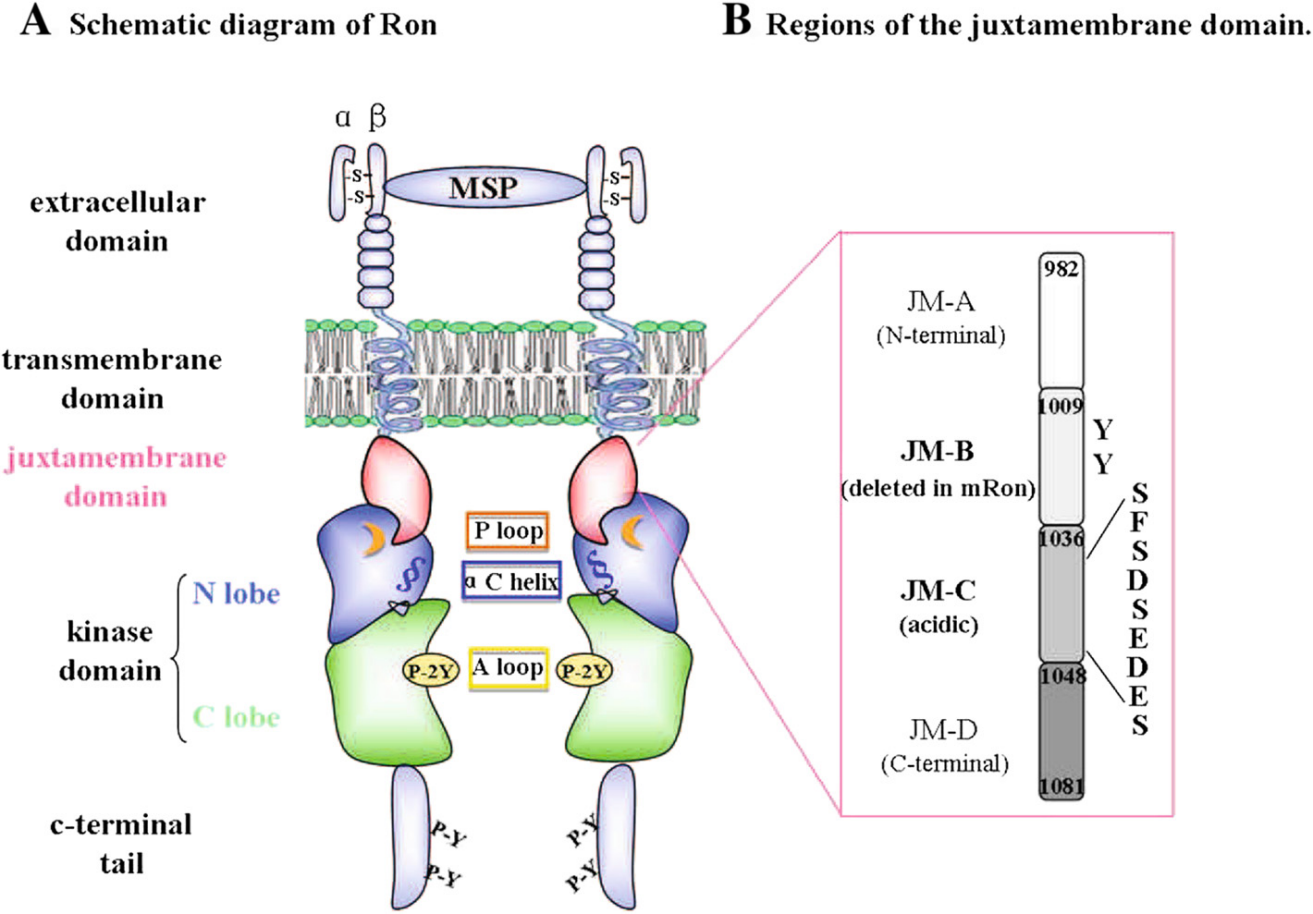
**Schematic diagram of the Ron receptor. A)** Domains of the Ron receptor. P loop (orange) and αC helix (blue) in the kinase N lobe and activation loop (yellow) in the C lobe are denoted by different colors. Critical tyrosines in the activation loop (P-2Y: Y^1238, 1239^) and c-terminal tail (P-Y: Y^1353,1360^) are indicated. **B)** The juxtamembrane domain of Ron is divided into four sequential regions: JM-A (residue 982–1008), the region at the N terminus; JM-B (1009–1035), the region corresponding to the sequence deleted in mRon; JM-C (1036–1047), the region with an acidic patch of residues; and JM-D (1048–1081), the region at the c-terminus of the juxtamembrane domain. Residues discussed herein are indicated.

Recepteur d'originenantais (Ron) (called STK in mice and Sea in chickens) belongs to the Met proto-oncogene family [19,20]. Originally synthesized as a single chain precursor, Ron is processed into a disulfide-linked heterodimer with a transmembrane β chain and an extracellular α chain [21,22]. Binding of macrophage stimulating protein (MSP), the ligand for Ron, to the extracellular domain of Ron induces receptor dimerization, conferring catalytic activity to the receptor [23]. The intracellular region of Ron includes the juxtamembrane domain, the highly conserved kinase domain and the non-catalytic c-terminal tail. The kinase domain consists of two lobes. The N-lobe contains the αC helix and the P loop which are essential for ATP recruitment, while phosphorylation of two tyrosines (Y^1238^ and Y^1239^) in the C-lobe activation loop is required for receptor activation. Subsequent phosphorylation of two docking tyrosines in the c-terminal tail transduces a variety of signaling pathways mediated by Ron [22,24] (Figure 1A).

The juxtamembrane domain, which forms an important layer of constraint, limiting promiscuous enzymatic activity of RTKs, is found frequently mutated in tumor cells [25-32]. The regulatory mechanisms mediated by the juxtamembrane domain however, varies significantly due to the varied length and lack of sequence similarity among RTKs. Murine Ron harboring a deletion of 27 residues in the juxtamembrane domain, caused by exclusion of a potential exon, exhibits significantly higher receptor activity than human Ron [33-36]. Deletion of the 27 residues in human Ron promotes receptor activation, highlighting a potential role for the juxtamembrane domain in the regulation of the Ron receptor activation [33]. However, the molecular mechanisms underlying this regulation are unknown.

Herein, we have characterized the mechanism by which the juxtamembrane domain contributes to the auto-regulation of Ron activity through structure/function analysis. While tyrosines in the Ron juxtamembrane domain are inconsequential for receptor activation, an acidic JM-C region in the juxtamembrane domain was found to be critical for Ron autoinhibition. Phosphorylation of Y^1198^ in the kinase domain promoted Ron activation, primarily by accelerating relief of the autoinhibition imposed by the JM-C region. Based on our experimental data, we have proposed molecular models of the inactive and the active Ron structure, which cast a new light on the mechanisms controlling Ron mediated signal transduction.

## Results

### The acidic JM-C region in the Ron juxtamembrane domain plays a critical role in receptor autoinhibition

The juxtamembrane domain of Ron is significantly longer than that of most RTKs, therefore we divide it into four sequential regions for clarity (Figure 1B). Previous studies have mapped the functional difference between murine Ron and human Ron to the 27 residues comprising the JM-B region, which contains Y^1012^ and Y^1017^, the only two tyrosines in the juxtamembrane domain (Additional file 1: Figure S1A). Tyrosines in the juxtamembrane domain of RTKs such as the EphR, Flt3 and Kit [29,32,37] are critical for maintenance of the inactive kinase, and phosphorylation of these tyrosines relieves receptor autoinhibition. However, consistent with previous reports [33,38], mutation of Y^1012^ and Y^1017^ individually or in combination into phenylalanine, glutamic acid or alanine in the context of Ron did not affect receptor autophosphorylation, downstream Erk phosphorylation (Additional file 1: Figure S1B), or induction of AP1 luciferase activity (Additional file 1: Figure S1C). Thus, unlike other RTKs, tyrosines in the juxtamembrane domain of Ron do not regulate receptor activation.

**Figure 2:**
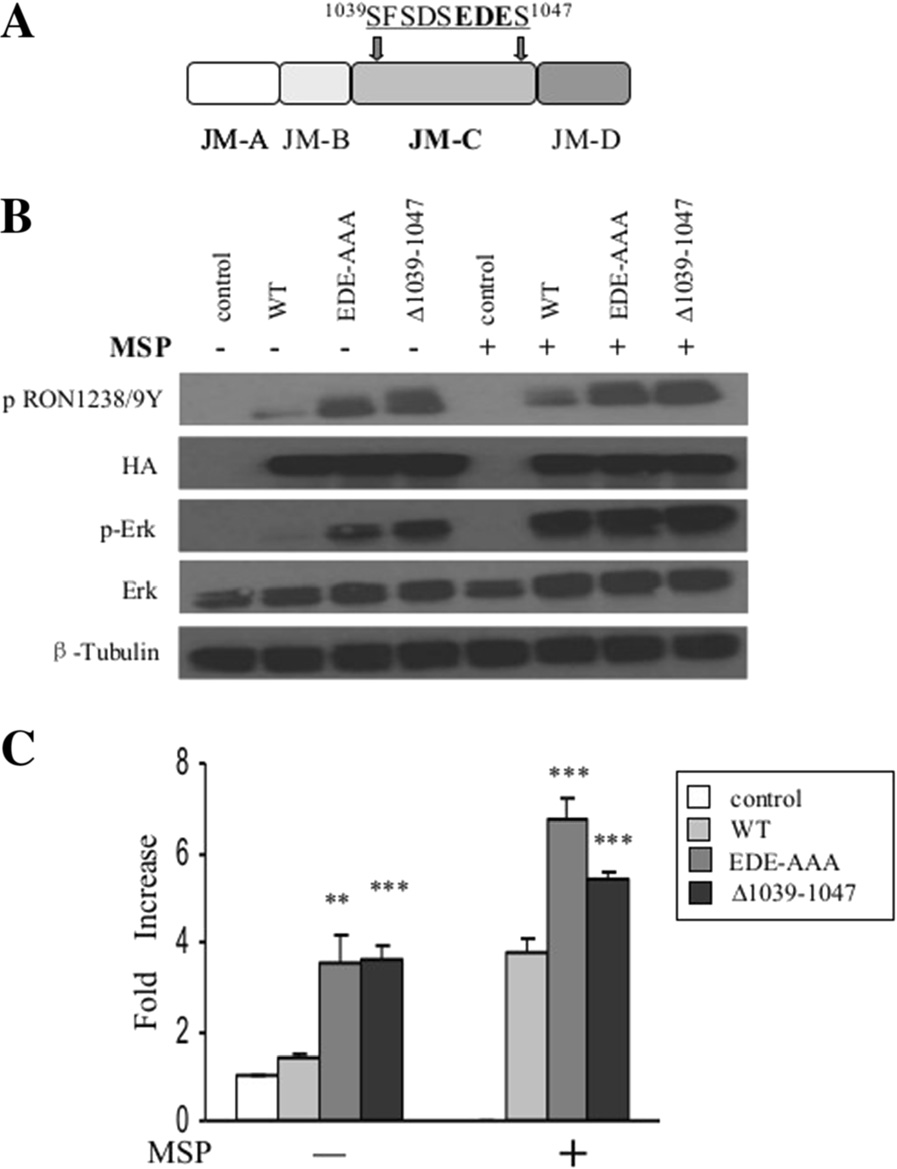
**Acidic residues in the JM-C region of Ron promote receptor autoinhibition. A)** Schematic of mutants. Mutations are highlighted in bold. Residues deleted are underlined. **B)** 293 cells were transfected with HA-tagged wild-type or mutant Ron as indicated in the presence and absence of MSP. Lysates were blotted for p-Ron1238/9Y, HA, p-Erk, Erk and β-tubulin. **C)** Relative luciferase activity in 293 cells co-transfected with wild-type or mutant Ron as indicated, and an AP-1 luciferase reporter in the presence and absence of MSP. The data were presented as the average ± standard deviation from measurements collected from three independent experiments and three measurements per experiment. Wild type Ron −/+MSP was used as controls, unless denoted with ‘︹’. The significant differences of values at *p* < 0.05, *p* < 0.01, and *p* < 0.001 levels are indicated by *, **and ***respectively.

While the primary sequence differs, acidic residues (glutamic acid and aspartic acid) interspersed with serines in the Ron JM-C region (Figure 2A) are relatively conserved in the closely related Met receptor, suggesting that this region might serve a conserved function. In order to determine the potential role of this region in the regulation of Ron receptor activity, we constructed a Ron mutant in which nine residues (most acidic) in the JM-C region were deleted (Ron^∆1039–1047^) (Figure 2A). HA-tagged wild-type Ron and Ron variants were transiently transfected in HEK 293 cells. Phosphorylation of the activation loop tyrosines (Y^1238/9^), a hallmark of kinase activation, and induction of a major signaling cascade downstream of Ron, the Ras-Map kinase pathway, were examined. Ron and the variants were also transiently transfected with an AP-1 luciferase reporter, and the induced AP-1 transcriptional response was measured. Equivalent levels of protein expression and cell surface expression of Ron and its variants were demonstrated by probing for HA and by the flow cytometry analysis (Additional file 2: Figure S2), respectively. Compared with the wild type receptor, Ron^∆1039–1047^ exhibited a significant increase in receptor autophosphorylation, induced Erk phosphorylation (Figure 2B) and AP-1 transcriptional activity both in the presence and absence of MSP (Figure 2C). In order to further determine whether the negative electrostatic nature of the JM-C region is responsible for its role in the regulation of Ron activity, E^1044^, D^1045^ and E^1046^ in the JM-C region were mutated to alanine individually and in combination. Single mutants did not show remarkable catalytic differences compared with wild type Ron (data not shown). However, the triple mutation (EDE-AAA) dramatically upregulated receptor activity and downstream signaling to a level comparable to that induced by Ron^∆1039–1047^ (Figure 2B and C). Based on these observations, we have identified a novel segment in the juxtamembrane domain of Ron, the JM-C region, which plays a central role in kinase autoinhibition, primarily mediated by the acidic residues.

**Figure 3:**
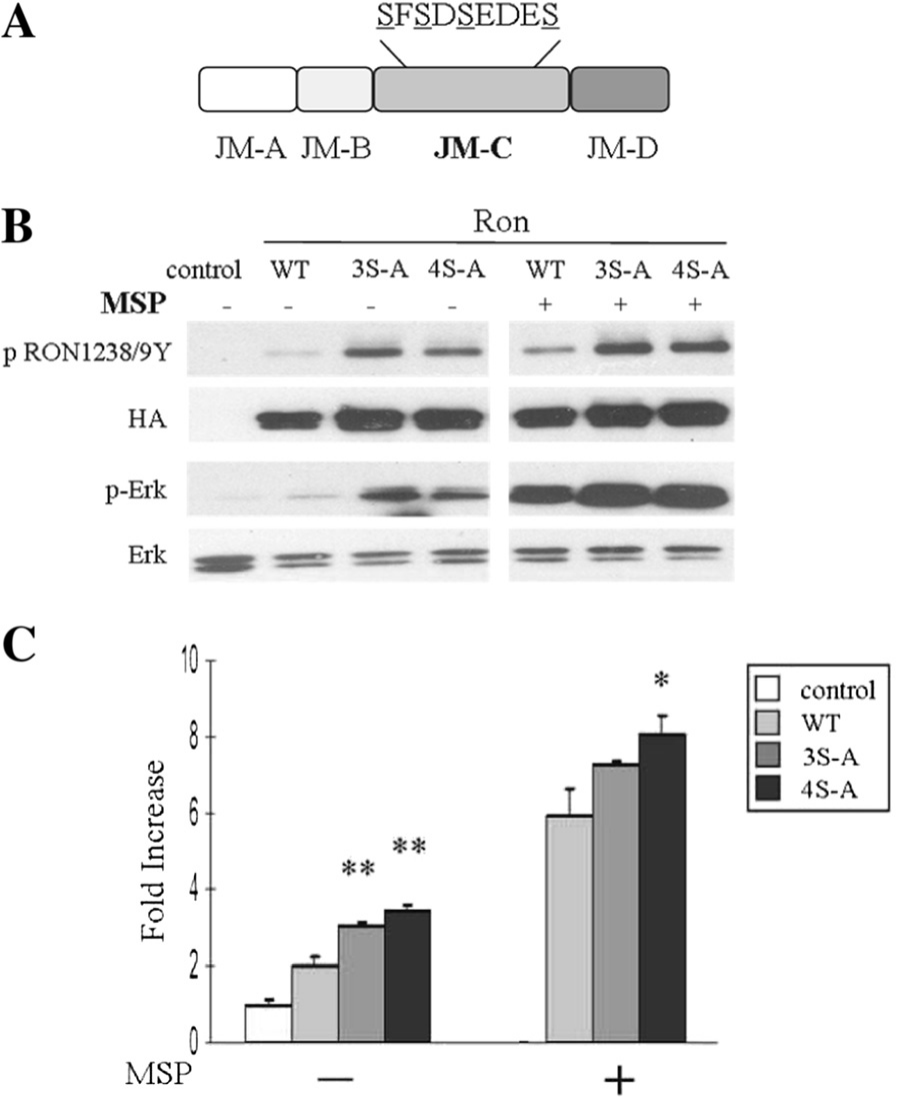
**Serines in the JM-C region play a subsidiary role in the regulation of Ron receptor activation. A)** Schematic of mutants. Mutations are highlighted by underline. **B)** 293 cells were transfected with HA-tagged wild-type or mutant Ron as indicated in the presence and absence of MSP. Lysates were blotted for p-Ron1238/9Y, HA, p-Erk and Erk. **C)** Relative luciferase activity in 293 cells co-transfected with wild-type or mutant Ron as indicated, and an AP-1 luciferase reporter in the presence and absence of MSP.

Serines (S^1039^,S^1041^,S^1043^,S^1047^) are major components of the acidic JM-C region, the first three of which are predicted to be potential phosphorylation sites (NetPhos 2.0 Server) (data not shown) (Figure 3A). In order to determine whether phosphorylation of these serines could play a role in the regulation of Ron receptor activity by the JM-C region, we mutated the serines to alanines individually and in combination. While individual and double mutants performed similarly as wild-type Ron (data not shown), the triple and quadruple mutations (3S-A and 4S-A) resulted in an evident increase in receptor activation and downstream signaling (Figure 3B and C). However, this activation was not as striking as that driven by mutations of the acidic ^1044^EDE residues. Thus, while the acidic residues in the JM-C region maintain the primary inhibitory constraints, phosphorylation of serines in this region may also contribute to the Ron autoinhibition mediated by the JM-C region.

### The JM-B region regulates receptor activity indirectly through its impact on the spatial orientation of the JM-C constraint

**Figure 4:**
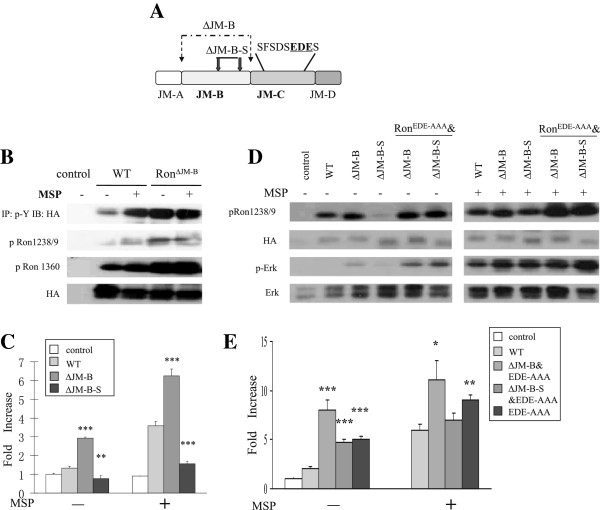
**The JM-B region modulates Ron receptor activation by affecting the orientation of the JM-C region. A)** Schematic of mutants. Deletions of different lengths in the JM-B region are indicated by arrows. Mutated residues are highlighted in bold. **B)** &**D)** 293 cells were transfected with HA-tagged wild-type or mutant Ron in the presence and absence of MSP. Lysates were immunoprecipitated with anti p-Y and blotted for HA, or directly blotted for p-Ron1238/9, p-Ron1360 and HA, p-Erk and Erk. **C)** &**E)** Relative luciferase activity in 293 cells co-transfected with wild-type or mutant Ron, and an AP-1 luciferase reporter in the presence and absence of MSP.

Previous studies demonstrated that deletion of the JM-B region of human Ron (P^1009^-V^1035^), absent in murine Ron, resulted in elevated receptor activity, as observed in murine Ron [33]. However, our studies suggest that individual residues in the JM-B region including tyrosines, are dispensable for Ron activation. Therefore, we reasoned that the JM-B region might play a structural role in Ron autoinhibition. Consistent with previous studies, here we found that Ron^∆JM-B^ possessed higher intrinsic catalytic capability than the wild type receptor, as demonstrated by a dramatic increase in phosphorylation of the activation loop tyrosines and a docking tyrosine, and in expression of an AP-1 reporter in both the presence and the absence of ligand (Figure 4B and C). In order to determine the significance of the length of the JM-B region, a Ron variant with a smaller deletion of ten residues (G^1020^-L^1029^) in the JM-B region, Ron^∆JM-B-S^, which did not result in deletion of the tyrosines in the JM-B region, was constructed (Figure 4A). Surprisingly, while Ron^∆JM-B^ exhibited enhanced receptor activity, Ron^∆JM-B-S^ exhibited decreased receptor activity, and the induced AP-1 transactivation was significantly reduced even in the presence of MSP (Figure 4C and D). These data indicate that the length of the JM-B region, rather than individual residues, appears to be a critical determinant for the proper regulation of Ron activity.

Since the acidic JM-C region lies c-terminal to the JM-B region of Ron, we hypothesized that the JM-B region might modulate receptor activity indirectly by affecting localization of the JM-C region. In order to test this hypothesis, the EDE-AAA mutation in the JM-C region was generated in the context of Ron^∆JM-B^ and Ron^∆JM-B-S^ (Figure 4A). Since both ∆JM-B and EDE-AAA were able to relieve Ron autoinhibition, as expected, Ron^∆JM-B^&^EDE-AAA^ exhibited enhanced catalytic activity both in the presence and absence of MSP (Figure 4D and E). However, the levels of AP-1 luciferase activity induced by Ron^∆JM-B^&^EDE-AAA^ were similar to those induced by Ron^EDE-AAA^, suggesting that the deletion of JM-B region and neutralization of the acidic JM-C region may facilitate receptor activation in an analogous manner. Alternatively, although the ∆JM-B-S deletion prohibited receptor activation, introduction of the EDE-AAA mutation into the Ron^∆JM-B-S^ construct largely restored receptor activity (Figure 4D and E). Thus, it is likely that the JM-B region modulates activation of the Ron receptor by affecting the spatial orientation of the adjacent JM-C region, which places autoinhibitory constraints on the kinase due to its electronegative characteristics.

### Y^1198^ in the kinase domain mediates Ron autoinhibition imposed by the JM-C region

**Figure 5:**
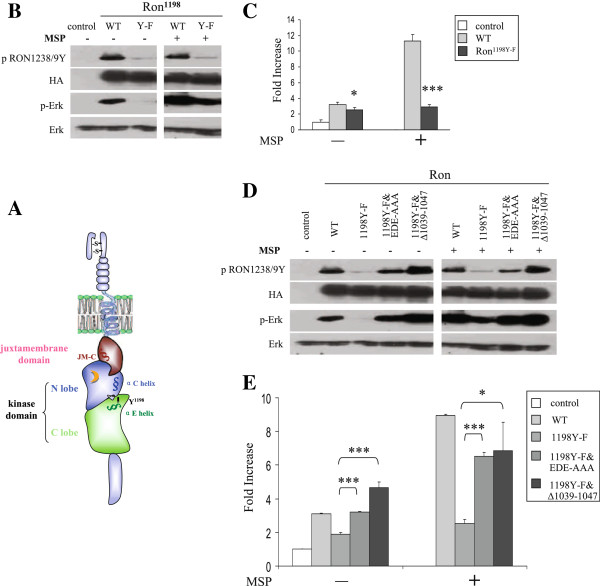
**Y**^**1198 **^**in the kinase domain of Ron regulates the autoinhibitory constraints on receptor activation imposed by the JM-C region. A)** Schematic of the Ron receptor. Y^1198^ at the interface of the αE helix and the αC helix is highlighted. **B)** &**D)** 293 cells were transfected with HA-tagged wild-type or mutant Ron as indicated in the presence and absence of MSP. Lysates were blotted for p-Ron1238/9Y, HA, p-Erk and Erk. **C)** &**E)** Relative luciferase activity in 293 cells co-transfected with wild-type or mutant Ron as indicated, and an AP-1 luciferase reporter in the presence and absence of MSP.

Previous studies indicate that Y^1194^ in Met, the equivalent residue of Y^1198^ in Ron, is one of the three major receptor autophosphorylation sites, and mutation of Y^1194^ to phenylalanine in Met results in dramatically decreased receptor activity [39,40]. Here we show that the equivalent Y1198F mutation in Ron dramatically reduced Ron autophosphorylation, as well as induction of downstream Erk phosphorylation and AP-1 transcriptional activation both in the presence and absence of MSP (Figure 5B and C). These results indicate that phosphorylation of Y^1198^ in the αE helix of the kinase C lobe plays a key role in the activation of wild type Ron. Although Y^1198^ and the juxtamembrane domain are unlikely to sterically interact with each other based on their distance, the potential functional interrelation was evaluated by introduction of the Y1198F mutation in the context of Ron^EDE-AAA^ and Ron^∆1039–1047^ (Figure 5A). Alterations in the JM-C region, which were demonstrated to relieve Ron autoinhibition, successfully rescued the loss of receptor activity caused by the 1198Y-F mutation. The double mutants recovered receptor activity to a level comparable to wild type Ron, and responded to stimulation by MSP (Figure 5D and E). These studies suggest that phosphorylation of Y^1198^ may play an essential role in relieving the inhibitory constraints on Ron activation imposed by the JM-C region in an indirect manner.

## Discussion

Mutations in the juxtamembrane domain are a common mechanism by which RTKs become constitutively activated and drive progression of a variety of human cancers. Internal tandem duplications in the juxtamembrane domain of Flt3 have been identified in patients with acute myeloid leukemia [41]. Deletions in the juxtamembrane domain of both KIT [42] and PDGFR [28] have been identified in human gastrointestinal stromal tumors. Germ line point mutations in the juxtamembrane domain of Met have been identified in small cell lung cancer [27] and gastric cancer [43]. An alternatively spliced form of Met, missing exon 14 which encodes part of its juxtamembrane domain [44], exhibits a highly tumorigenic phenotype [31,45]. Likewise, a splice variant of RON, missing exon 14 (BG289902) in the juxtamembrane domain, was found in a bladder papilloma cell line (human Expressed Sequence Tag data base). The juxtamembrane domain of RTKs varies in length and lacks apparent sequence similarity, suggesting that it may contribute to specific aspects of regulation within individual receptor families. Our studies demonstrate that, like other RTKs, the juxtamembrane domain of Ron plays a central role in receptor autoinhibition. However, the mode of regulation conferred by the juxtamembrane domain of Ron appears to be novel compared with other RTKs described to date.

Tyrosine phosphorylation in the juxtamembrane domain of RTKs including Kit, Flt3 and EphR, is a common mechanism by which the receptor switches from the default closed state to an active configuration [29,32,37,46]. However, phosphorylation and the aromatic side chain of tyrosines in the JM-B region are dispensable for Ron activity. Conversely, deletions of different lengths in the JM-B region were found to have significant but opposite impacts on receptor activation, probably through affecting the spatial orientation of the contiguous JM-C region.

**Figure 6:**
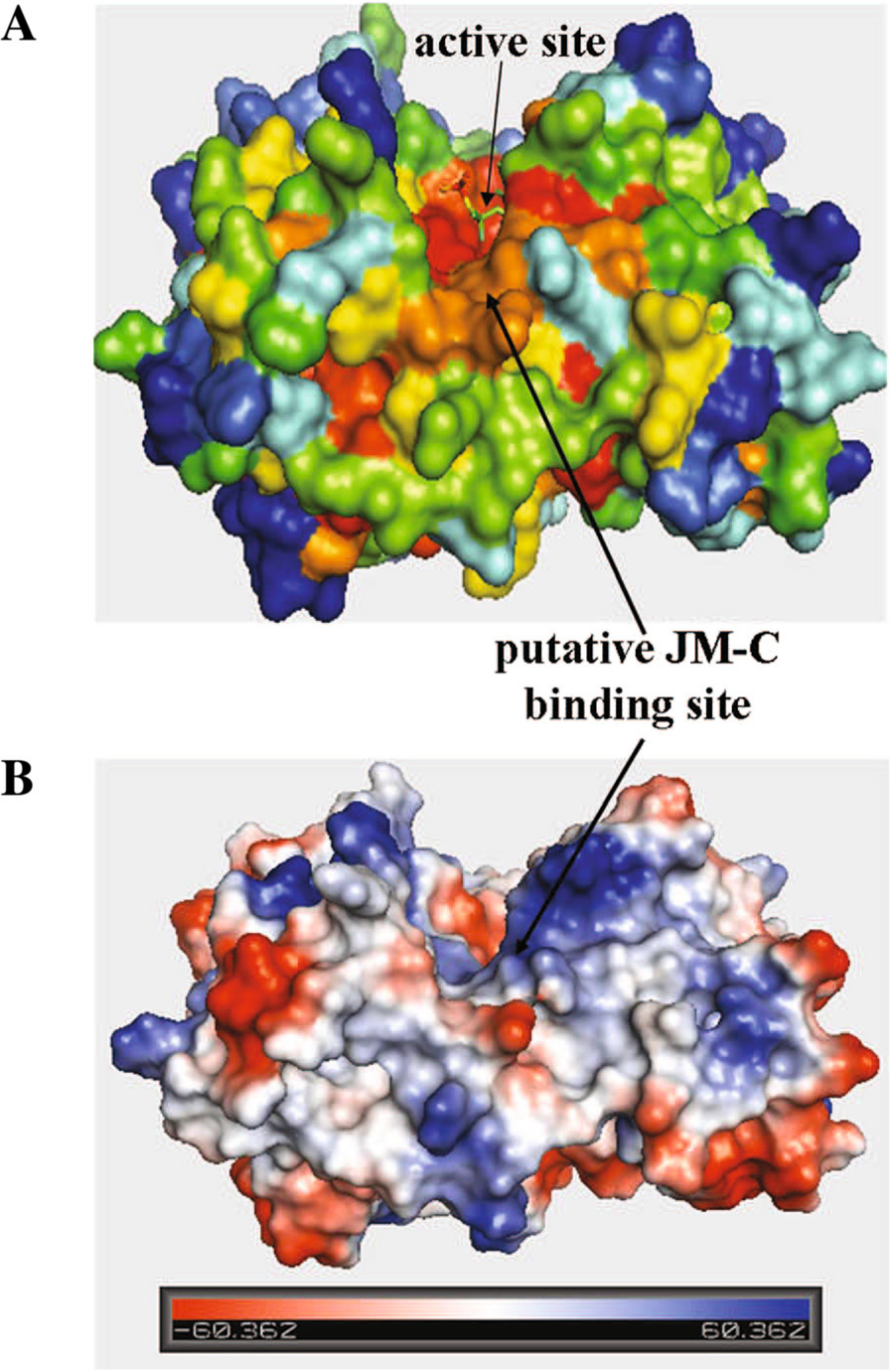
**Conserved surface residues and surface potential of the Ron kinase domain (3PLS) [**[48]**].** The conserved region adjacent to the active site with a predominantly positive surface potential is indicated by arrows as the putative JM-C binding site. **A)** Conserved residues on the surface of human kinase domains are indicated, with red/orange representing highly conserved residues and blue/cyan representing highly variable residues. **B)** Surface potential of the Ron kinase domain, with blue representing positive potential and red representing negative potential. The units of the scale were −60.362 ~ 60.362.

Here, we show that the acidic cluster in the JM-C region of Ron is a crucial regulator of receptor activity, and disruption of its negative electrostatic property dramatically facilitates kinase activation. The acidic characteristics of the JM-C region are relatively conserved within the Met family members. The equivalent region in Met was found to contain a mutation (S1058P) in a sample from the non small cell lung cancer [47], suggesting the potential functional significance of this region. In order to better understand the inhibitory mechanism imposed by the JM-C region, we compared the kinase domain sequences of 150 receptors by mapping them onto the crystal structure of the Ron kinase domain (3PLS) [48] (Figure 6). Based on surface accessibility and relative location, a conserved region on the surface of the Ron kinase N lobe was identified as a putative JM-C binding region (orange in Figure 6A). It locates adjacent to the strictly conserved active site (red in Figure 6A), with a predominantly positive surface potential (blue in Figure 6B).

Based on the crystal structure of the Ron kinase domain, we built a molecular model of the inactive Ron structure (including JM-C, JM-D region and kinase domain) [48-51] (Additional file 3: Figure S3A), which suggests that the JM-C region (magenta) promoting a helical conformation could make close contacts with the P loop (orange) and activation loop (yellow). Acidic residues in the JM-C region (E^1044^-D^1045^-E^1046^) have the potential to interact with H^1092^ in the P-loop, Y^1238^ and Q^1243^ in the activation loop, and the side-chains of S^1117^ and R^1118^ between the β3 strand and the αC helix (lines in Additional file 3: Figure S3A). These interactions would interfere with the accommodation of ATP and substrates by the glycine rich P-loop. Spatial proximity of the JM-C region could also lock the activation loop in the inter-lobe cleft and prevent productive rotation of the N and C lobe. In addition, the rigid configuration promoted by the JM-C region, of the β3 strand and αC helix in the kinase N lobe, puts the invariant K^1114^ and E^1130^ that participate in formation of a salt bridge required for ATP binding in catalytically disfavored positions.

Located at the interface between the αE and the αC helix, Y^1198^ of Ron is highly conserved among RTKs and shares structural conservation among close relatives including Met, Ret, the insulin receptor, IGF1R and FGFR [40,52-55]. Phosphorylation of the equivalent tyrosine in Met and murine Ron is closely associated with receptor activity [36,39,40]. In the crystal structure of the Ron kinase domain [48], the nonproductive orientation of the αC helix is held by two hydrogen bonds (Additional file 4: Figure S4). One is between the side chain hydroxyl of Y^1198^ in the αE helix and the backbone amide of N^1139^ at the C-terminus of the αC helix, and the other one is between R^1231^ and Q^1124^ at the N-terminus. In order to further understand the events leading to receptor activation, we modeled the activated Ron kinase domain based on the crystal structure of active Met (3Q6U) [56] (Additional file 3: Figure S3B). In this model, the hydrogen bond between Y^1198^ and N^1139^ is disrupted, potentially due to the phosphorylation of Y^1198^. Consequently, the regional stability of the inactive kinase domain collapses, and the αC helix reorients significantly compared to its position in the auto-inhibited model. This large scale movement could promote the displacement of the JM-C constraint, resulting in reorientation of the P-loop and activation loop, and realignment of the active site residues for recruitment of ATP and substrates. Therefore, when Y^1198^ is mutated to phenylalanine, the absence of phosphorylation at this residue would be predicted to fix the αC helix in the kinked non-productive orientation, stabilizing the kinase in the inactive state. However, when the inhibitory JM-C region is released by alterations in the juxtamembrane domain that disrupt the autoinhibition confered by the juxtamembrane domain, phosphorylation of Y^1198^ would not be required for reorientation of the kinase segments and receptor activation.

## Conclusions

Because the mode of regulation by the juxtamembrane domain varies significantly among RTKs, targeting this tier of regulation could provide an opportunity to develop inhibitors that would exploit the unique aspects of juxtamembrane domain regulation conferred upon individual receptors. In that context, a synthesized peptide representing the juxtamembrane domain of Kit is able to impede receptor activation *in vitro* and delay growth of cells containing an oncogenic form of Kit [37]. Alternatively, targeting the autoinhibited form of the receptor with small molecule inhibitors could provide an additional level of specificity due to the association between the juxtamembrane domain and the active site. The work presented here has unveiled a novel regulatory mechanism governing Ron activity by its juxtamembrane domain, and provided a new perspective regarding the regulation of RTK activity through conformational plasticity and functional cooperation of different protein segments, which may provide important insights that could lead to the development of a novel class of therapeutics for the treatment against a wide range of Ron mediated carcinomas.

## Methods

### Gene construction and mutagenesis

Human RON and mutants were expressed by the mammalian pcDNA3.1 vector or the murine stem cell virus (MSCV) retroviral vector. Mutants were generated from pcDNA-RON-HA or MSCV-RON-HA, with the Quik Change mutagenesis kit (Stratagene) according to the manufacturer’s instructions. The following PCR protocol was used and primers for the mutants are listed in Additional file 5: Table S1: 95°C 30 s, followed by 95°C 30 s, 55°C 1 min, 68°C 12 min for 20 cycles, and a final 68°C 12 min.

### Cell culture, antibodies, and reagents

Human embryonic kidney (HEK) 293 cells (ATCC) were maintained in Dulbecco’s modified Eagle’s medium supplemented with 10% fetal bovine serum. The TransIT-293 transfection reagents were purchased from Mirus Bio, LLC (Madison, WI). The dual-luciferase reporter assay system was purchased from Promega Corporation (Madison, WI). Antibodies against Ron 1238/9phospho-tyrosine, phospho-Erk, Erk and the HA epitope (262 K) were purchased from Cell Signaling. Goat anti-rabbit IgG-HRP was purchased from Santa Cruz Biotechnology, Inc (Santa Cruz, CA). Recombinant MSP was purchased from R&D Systems. The AP-1 luciferase reporter cDNA was kindly provided by Dr. Avery August (Cornell University). All PCR primers were ordered from Eurofins MWG Operon, Inc. (Huntsville, AL). Pfu Turbo DNA polymerase was purchased from Agilent Tech. ECL Plus Western blotting detection reagents were purchased from GE Healthcare (Piscataway, NJ).

### Cell transfection and luciferase assays

For the luciferase assay, 5 × 10^4^ HEK 293 cells/well were seeded into 24-well plates. Twenty four hours later, a designated mixture of 60 ng of wild type or mutant forms of RON, 60 ng of AP-1 luciferase reporter plasmid, and 0.5 ng renilla were used for transient transfection with the Mirus-293 transfection reagent according to the manufacturer’s protocol. MSCV-neo or PCDNA3.1-neo plasmid were used as control vectors for each transfection. Cells were stimulated with 50 ng/ml (50 ug/ml stock) MSP 24 h following transfection. Luciferase assays were performed at 24 h later according to the manufacturer’s instructions (Promega). The fold increase is calculated as the firefly luciferase activity divided by the renilla luciferase activity.

### Western blot analysis

A total of 2.5 × 10^5^HEK 293 cells/well were plated into six-well plates. Twenty four hours later, the cells were transiently transfected with 300 ng RON or its derivatives per well. Cells were stimulated with 200 ng/ml MSP for 48 h following transfection. 10 minutes later, cells were suspended in 400 ul ice-cold cell lysis buffer containing 150 mM NaCl, 20 mM Tris–HCl (pH 7.5), 5 mM EDTA, 1% NP-40, 1 mM phenylmethylsulfonyl fluoride, 1 mM Na_3_VO_4_, and 10 mM NaF. Cell lysates were centrifuged at 12,000 rpm for 20 min, and the supernatant lysates were transferred to prechilled tubes. Cell lysates were mixed with 4X denaturing SDS loading buffer and heated at 100°C for 8 min. Samples were resolved on an SDS-PAGE gel and then transferred to polyvinylidene difluoride membranes. Membranes were then blocked with 5% nonfat milk or Bovine Serum Albumin in Tris-buffered saline with 0.1% Tween 20 (TBST) for 1 h and probed with primary antibody at 4°C overnight. Membranes were washed three times in TBST and incubated with secondary goat anti-rabbit IgG-HRP for another hour. Membranes were washed three times in TBST before ECL plus Western blotting detection reagents were applied for visualization. For reprobing, membranes were stripped with 62.5 mM Tris–HCl (pH 6.8), 2% SDS, and 0.7% β-mercaptoethanol at 55°C for 30 min.

### Flow cytometry

A total of 1.2 × 10^5^ HEK 293 cells/well were plated into 12-well plate. Twenty four hours later, the cells were transiently transfected with 200 ng RON or its derivatives per dish. PCDNA3.1-neo plasmid was used as control vectors for each transfection. Flow cytometry analysis was performed at 48 h later. 10^6^ cells were incubated per tube in 50 ul FACS buffer (ice cold PBS + 2% FBS). Non-specific binding was blocked by the addition of 50 μL HAB for 2 minutes at room temperature. Cells were incubated on ice with 5 ug/ml anti-Ronβ (DX07) or IgG (control) for 30 min. Cells were washed twice with 1 ml FACS buffer and resuspended once more in 50 μL of FACS buffer. 50 μL HAB per tube was added again for blocking of non-specific binding. Cells were incubated with 5 ug/ml PE anti-mouse IgG_1_ as a secondary antibody to Ron for 30 min on ice. Cells were washed twice, resuspended in 1 mL of FACS buffer and analyzed for FLOW on a Beckman-Coulter FC500.

### Evaluation of surface conservation and potential

A comparison of 150 receptor kinase domain sequences was mapped onto the crystal structure of the Ron kinase domain (PDB code 3pls) using the CONSURF web server. Surface conservation and surface potential of the Ron kinase domain were illustrated. Potential JM-C binding site was proposed based on residue/sequence characteristics and relative distance.

### Molecular modeling of the JM-C region

Modeling of the autoinhibited and the activated conformation of Ron was based on the crystal structure of the kinase domains of Ron (PDB code 3PLS) and Met (PDB code 3Q6U) respectively. The modeling was done manually using the COOT software [49]. Hydrogen bonds between side chains were manually optimized and the resultant model was energy minimized by using the Yasara server [51].

### Structure-based sequence analysis

Seven algorithms, BPS [16], D_R [57], DSC [58], GGR [59], GOR [60], G_G [61], H_K [62], K_S [63], L_G [64], and Q_S [65], were used for secondary structure prediction. The Joint prediction (JOI) is the prediction made by the program that assigns the structure using a “winner takes all” approach.

## Competing interests

The authors declare that they have no competing interests.

## Authors’ contributions

XW performed all the experiments and wrote the manuscript, NY performed all of the molecular modeling, PAH designed the experiments and helped write the manuscript. All authors read and approved the final manuscript.

## Supplementary Material

Additional file 1: Figure S1 Tyrosines in the juxtamembrane domain of Ron are dispensable for the regulation of receptor activity. **A)** Schematic diagram of mutants. Y^1012^ and Y^1017^ in the JM-B region are highlighted in bold. **B)** 293 cells were transiently transfected with HA-tagged wild-type or mutant Ron in the presence and absence of MSP. Lysates were blotted for p-Ron1238/9, HA, p-Erk and total Erk. **C)** Relative luciferase activity in 293 cells co-transfected with wild-type or mutant Ron, and an AP-1 luciferase reporter in the presence and absence of MSP. Click here for file

Additional file 2: Figure S2 Cell surface expression of wild type Ron and the Ron variants discussed herein. 293 cells were transiently transfected with wild-type or mutated Ron as indicated. Cells were harvested 48 h after transfection and analyzed by flow cytometry for protein membrane expression. Click here for file

Additional file 3: Figure S3 Predicted structure of the autoinhibited and active form of Ron. **A)** In the inactive Ron model, the JM-C region (magenta) is predicted to pack against and interact with the activation loop (yellow) and P-loop (orange) near the active site of the kinase domain. Potential bonds among residues in the JM-C region (^1044^EDE) (magenta), the P loop (H^1092^) (orange), the active site (S^1117^, R^1118^) (green) and the activation loop (E^1237^, Y^1238^) (yellow) are labeled with black dashed lines. Y^1198^ (cyan stick) in the kinase C-lobe hydrogen bonds to the backbone amide of N^1139^ at the c-terminus of the αC helix. **B)** In the active Ron model, phosphorylation of Y^1198^ results in reorientation of the αC helix and JM-D region. Consequently, the JM-C region is released from the active site, which enables ATP binding and reorientation of the activation loop. The orange and cyan stick represents AMP-PNP. Figure was generated using the software Pymol. Click here for file

Additional file 4: Figure S4 Y^1198^-N^1139^ hydrogen bond promotes the non productive orientation of the αC helix. Crystal structure of the autoinhibited Ron kinase domain (3PLS) is shown as cartoon. The αC helix and αE helix are colored in cyan and green respectively. Residues involved in the hydrogen bonds (yellow dash) linking the two helices are labeled in white. Click here for file

Additional file 5: Table S1 PCR primers for the mutant construction. Click here for file
