# Supplementary figures and images for: Autoinhibition of the Ron receptor tyrosine kinase by the juxtamembrane domain
Source: Cell Commun Signal. 2014 Apr 16;12:28. doi: 10.1186/1478-811X-12-28 (PMC4021555; doi:10.1186/1478-811X-12-28)

**A**

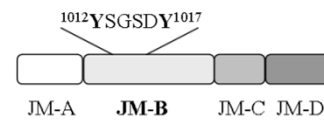

**B**

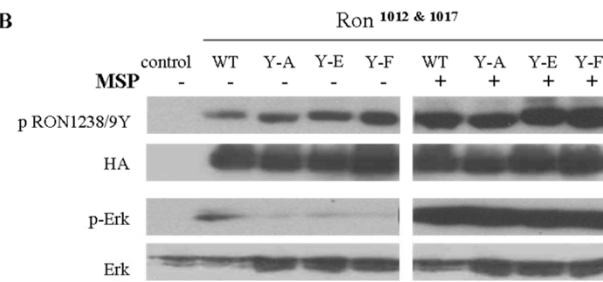

**C**

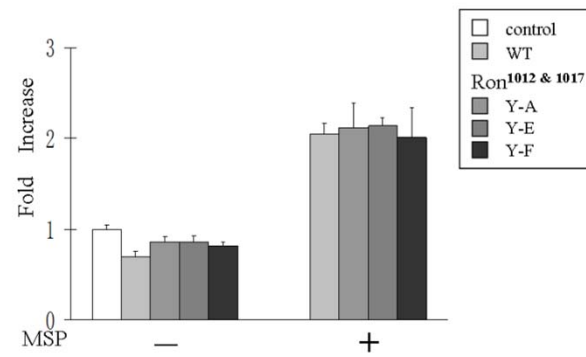

Supplement: Additional file 1: Figure S1 — Tyrosines in the juxtamembrane domain of Ron are dispensable for the regulation of receptor activity. A) Schematic diagram of mutants. Y1012 and Y1017 in the JM-B region are highlighted in bold. B) 293 cells were transiently transfected with HA-tagged wild-type or mutant Ron in the presence and absence of MSP. Lysates were blotted for p-Ron1238/9, HA, p-Erk and total Erk. C) Relative luciferase activity in 293 cells co-transfected with wild-type or mutant Ron, and an AP-1 luciferase reporter in the presence and absence of MSP. [file 1478-811X-12-28-S1.pdf]

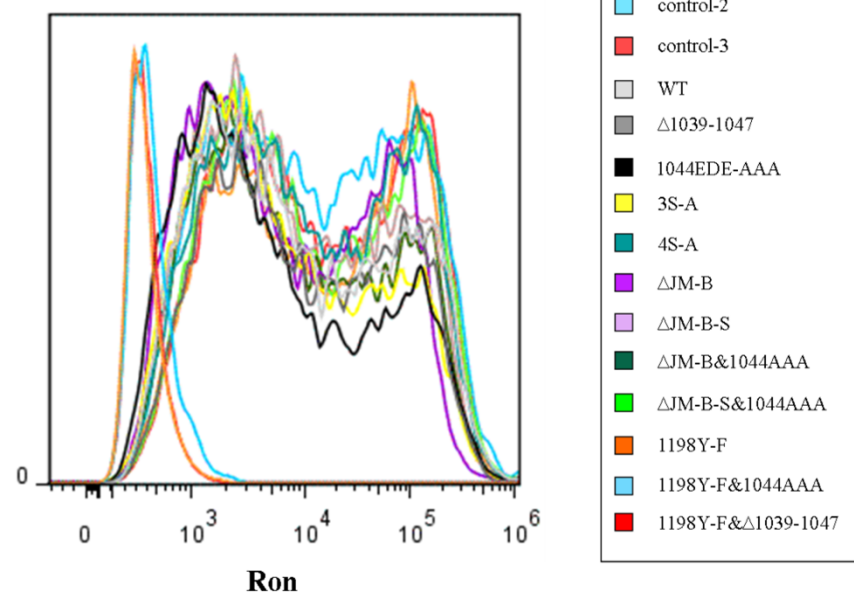

Supplement: Additional file 2: Figure S2 — Cell surface expression of wild type Ron and the Ron variants discussed herein. 293 cells were transiently transfected with wild-type or mutated Ron as indicated. Cells were harvested 48 h after transfection and analyzed by flow cytometry for protein membrane expression. [file 1478-811X-12-28-S2.pdf]

Figure S3

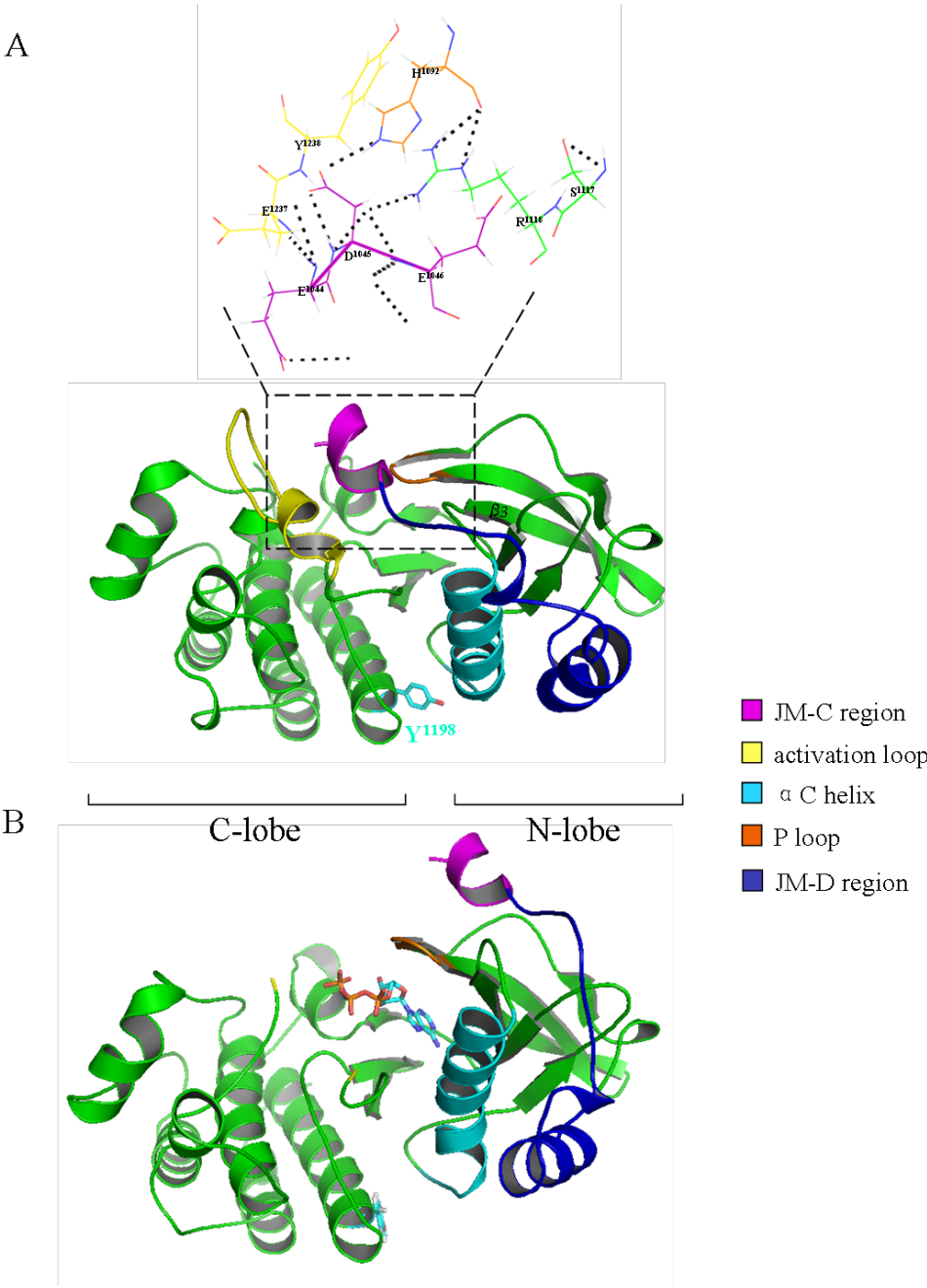

Supplement: Additional file 3: Figure S3 — Predicted structure of the autoinhibited and active form of Ron. A) In the inactive Ron model, the JM-C region (magenta) is predicted to pack against and interact with the activation loop (yellow) and P-loop (orange) near the active site of the kinase domain. Potential bonds among residues in the JM-C region (1044EDE) (magenta), the P loop (H1092) (orange), the active site (S1117, R1118) (green) and the activation loop (E1237, Y1238) (yellow) are labeled with black dashed lines. Y1198 (cyan stick) in the kinase C-lobe hydrogen bonds to the backbone amide of N1139 at the c-terminus of the αC helix. B) In the active Ron model, phosphorylation of Y1198 results in reorientation of the αC helix and JM-D region. Consequently, the JM-C region is released from the active site, which enables ATP binding and reorientation of the activation loop. The orange and cyan stick represents AMP-PNP. Figure was generated using the software Pymol. [file 1478-811X-12-28-S3.pdf]

Figure S4

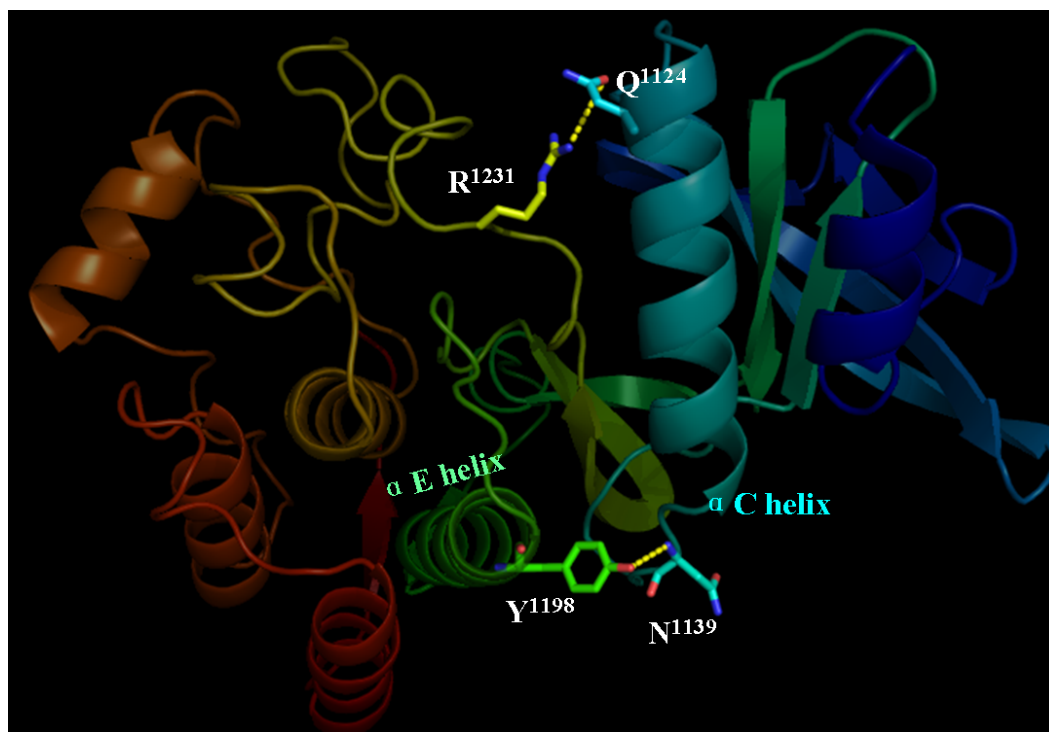

Supplement: Additional file 4: Figure S4 — Y1198-N1139 hydrogen bond promotes the non productive orientation of the αC helix. Crystal structure of the autoinhibited Ron kinase domain (3PLS) is shown as cartoon. The αC helix and αE helix are colored in cyan and green respectively. Residues involved in the hydrogen bonds (yellow dash) linking the two helices are labeled in white. [file 1478-811X-12-28-S4.pdf]
