# Supplementary material for: Autoinhibition of the Ron receptor tyrosine kinase by the juxtamembrane domain
Source: Cell Commun Signal. 2014 Apr 16;12:28. doi: 10.1186/1478-811X-12-28 (PMC4021555; doi:10.1186/1478-811X-12-28)
Supplement: Additional file 5: Table S1 — PCR primers for the mutant construction. [file 1478-811X-12-28-S5.pdf]

MUTANT	FORWARD PRIMER
$Ron^{1012Y\text{-}A}$	5’-CCCCTGCCTATTCTGGCCTCGGGCTCTGAC-3’
$Ron^{1012Y\text{-}E}$	5’-CCCCTGCCTATTCTGGAATCGGGCTCTGAC-3’
$Ron^{1012Y\text{-}F}$	5’-CCCCTGCCTATTCTGTTCTCGGGCTCTGAC-3’
$Ron^{1017Y\text{-}A}$	5’-GTACTCGGGCTCTGACGCCAGAAGTGGCCTTGC-3’
$Ron^{1017Y\text{-}E}$	5’-GTACTCGGGCTCTGACGAAAGAAGTGGCCTTGC-3’
$Ron^{1017Y\text{-}F}$	5’-GTACTCGGGCTCTGACTTCAGAAGTGGCCTTGC-3’
$Ron^{1013AGSAYA}$	5’-CCTGCCTATTCTGTACGCGGGCTCTGCCTACGCAAGTGGCCTTGC-3’
$Ron^{\Delta 1020\text{-}1029}$	5’-CTGACTACAGAAGTGATTCCACCACTTG-3’
$Ron^{1044E\text{-}A}$	5’-CCTTCTCCGATAGTGCAGATGAATCCTGTGTGCCAC -3’
$Ron^{1045D\text{-}A}$	5’-CCTTCTCCGATAGTGAAGCTGAATCCTGTGTGCCAC -3’
$Ron^{1046E\text{-}A}$	5’-CCTTCTCCGATAGTGAAGATGCATCCTGTGTGCCAC-3’
$Ron^{1044EDE\text{-}AAA}$	5’-CCTTCTCCGATAGTGCAGCTGCATCCTGTGTGCCAC-3’
$Ron^{3S\text{-}A}$	5’-GTCCATGGAGCAGCCTTCGCCGATGCTGAAGATGAATCC-3’
$Ron^{4S\text{-}A}$	5’-GAAGATGAAGCCTGTGTGCCACTGCTGCG-3’
$Ron^{1198Y\text{-}F}$	5’-CCGCGGCATGGAGTTCCTGGCAGAGCAG-3’
$Ron^{1254M\text{-}T}$	5’-CCTGTGAAGTGGACGGCGCTGGAGAGCC-3’
